# Numerical Simulation in the Melt Pool Evolution of Laser Powder Bed Fusion Process for Ti6Al4V

**DOI:** 10.3390/ma15217585

**Published:** 2022-10-28

**Authors:** Yixuan Xu, Dongyun Zhang, Junyuan Deng, Xuping Wu, Lingshan Li, Yinkai Xie, Reinhart Poprawe, Johannes Henrich Schleifenbaum, Stephan Ziegler

**Affiliations:** 1 Institute of Laser Engineering, Faculty of Materials and Manufacturing, Beijing University of Technology, Beijing 100124, China; 2 Engineering Research Center of 3D Printing at Beijing University of Technology, Pingleyuan No. 100, Chaoyang Dist, Beijing 100124, China; 3 Fraunhofer Institute for Laser Technology ILT, D-52074 Aachen, Germany; 4 RWTH Aachen University-Digital Additive Production (DAP), D-52074 Aachen, Germany

**Keywords:** laser powder bed fusion (L-PBF) process, numerical simulation, melt pool evolution, Ti6Al4V, fluid flow, temperature distribution

## Abstract

In order to track the free interface of the melt pool and understand the evolution of the melt pool, the flow of fluid, and the interface behavior of gas and liquid, a physical model is developed by using the VOF method in this paper. Its characteristics are a combined heat source model, including a parabolic rotation and a cylindrical distribution, and a powder bed stochastic distributed model with powder particle size. The unit interface between the metallic and gas phase in the laser–powder interaction zone can only be loaded by the heat source. Only the first and second laser scanning tracks are simulated to reduce the calculation time. The simulation results show that process parameters such as laser power and scanning speed have significant effects on the fluid flow and surface morphology in the melt pool, which are in good agreement with the experimental results. Compared with the first track, the second track has larger melt pool geometry, higher melt temperature, and faster fluid flow. The melt flows intensely at the initial position due to the high flow rate in the limited melt space. Because there is enough space for the metal flow, the second track can obtain smooth surface morphology more easily compared to the first track. The melt pool temperature at the laser beam center fluctuates during the laser scanning process. This depends on the effects of the interaction between heat conduction or heat accumulation or the interaction between heat accumulation and violent fluid flow. The temperature distribution and fluid flow in the melt pool benefit the analysis and understanding of the evolution mechanism of the melt pool geometry and surface topography and further allow regulation of the L-PBF process of Ti6Al4V.

## 1. Introduction

Additive manufacturing (AM) is an advanced manufacturing technology that innovatively transforms the conventional manufacturing of “material reduction” into that of “material addition”. It has the advantages of short processing cycle, high material utilization, and the ability to fabricate components with complicated geometry, as mentioned in [1,2], while providing the possibility to overcome the “bottleneck” in conventional production manufacturing.

Laser powder bed fusion (L-PBF, also known as SLM) is one of the most promising manufacturing methods of metallic components among AM technologies [3]. During the process, a high-power laser selectively irradiates and melts the powder particles according to the data to be fabricated in a CAD model. By overlapping from points to lines to planes of laser scanning tracks, a 3D component with full density is fabricated. The L-PBF process has the unique characteristics of ultrafast heating [4] and cooling rates (10^4^–10^5^ T/s), as mentioned in the literature [5], and the ultrashort existence time of the melt pool is in the order of microseconds. However, the laser–powder interaction is a complex physical, chemical, and metallurgical process [6]. It is difficult to observe the process of the melt pool evolution and obtain data on the temperature distribution and fluid flow. The above information is significantly determined by laser power, scanning velocity, and other process parameters, which in turn influence the melt pool characteristics. Therefore, the process of the melt pool evolution can be reconstructed by numerical simulation based on a simplified and reliable mathematical model, and dynamic information on the process of evolution that occurs within a few microseconds can be obtained. Considering the calculation efficiency and the accuracy of results, it is also an effective method to understand the L-PBF process [7].

The finite element method (FEM) is employed to simulate the L-PBF process to understand the characteristics of the melt pool, as mentioned in [8]. The volume of fluid (VOF) method, a useful tool for interface tracing in FEM, is also widely applied in research on the fluid flow in simulation [9], solidification and surface tension of the melt pool [10], and the mechanism of the melt pool evolution during the L-PBF process. Gürtler et al. [11] established a 3D model using OpenFOAM software with VOF to research the laser–material interaction. However, the powder bed model is based on a single particle size. It is well known that a powder bed model with a Gaussian distribution of powder particle size can truly reflect the complexity of the actual powder layer with particle packing. This causes the unsteady state of the fluid flow in the melt pool, compared with the steady fluid flow produced by the powder bed model simplified as a continuum body with the same thickness in [12] and with a single particle size. Tran and Lo [13] considered the depth of laser power penetration and built a new volumetric heat source for the size of the melt pool, which showed the effect of the heat source model on the processing simulation.

Boley et al. [14] traced the transmission of the laser beam on the powder bed and found that the substrate can absorb only a small amount of laser energy. Diffuse reflection plays an important role in the absorption of the powder layer to laser energy. This indicates that the energy absorption on the surface and inside the powder layer is different. At the same time, it suggests that different energy inputs at different positions of the powder layer should be considered in the development of a heat source in the simulation. Additionally, Zhang et al. [15] evaluated and compared eight commonly used heat source models and proposed a new 3D heat transfer finite element model. By verifying the molten pool size and track surface morphology, the obtained simulation results were in better agreement with the experimental molten pool size and track surface morphology. Therefore, it is necessary to choose an appropriate heat source in simulation.

Khairallah et al. [16] investigated the process of melt pool evolution in simulation with laser ray tracing heat source and powder bed models with stochastic particle size distribution. The results showed the importance of recoil pressure and Marangoni convection in the formation process of the melt pool, as well as the formation mechanism of denudation, splash, porosity, and other possible defects during the L-PBF process.

Wen et al. [17] established a finite element model (FEM) and implemented the properties of powders, molten pools, and solid metallic materials as a function of temperature. Compared with the experimental results, the model can simulate the temperature field distribution in the L-PBF process well. With the increase in laser power, the width and depth of the molten pool increase, and the cooling rate decreases; with the increase in scanning speed, the width and depth of the molten pool decrease, and the cooling rate increases. Shrestha et al. [18] used FLOW-3D to develop a physical model of the LPBF process, and the shape of the small hole caused by different laser power and scanning speeds was studied. The results showed that the pinhole size increases with laser power even at the same energy density. Cao et al. [19] predicted the evolution of pores at the mesoscale during the formation of LPBF based on the open-source computational fluid dynamics code OpenFOAM. By comparing the simulation and experimental results, it was concluded that when the volume energy density is too small, pore defects are generated due to insufficient fusion of metal particles; if the energy density is too large, the “keyhole” effect of the material is caused because the entrained gas cannot escape in time.

Khorasani et al. [20] pointed out that the surface morphology of the melt pool can be observed only by microscope after fabrication. There is not enough information to explain the formation mechanism of surface morphology due to the temperature distribution and fluid flow, which are decisive for the melt pool evolution. More and more simulations have revealed the formation mechanism of defects such as spatter [21], pores [22], and denudation [23] through research on the temperature distribution and fluid flow in the melt pool, which provide more details to better understand the L-PBF process reported by Leung et al. [24].

In short, previous research has simplified the powder bed as a continuum body with the same thickness or considered the laser scanning track to be a cylindrical weld for use in simulation in traditional welding technology. The heat source is a commonly used volume heat source, considering the heat not just acting on the surface but ignoring its difference in the surface of and inside the powder layer. An innovative model with a new heat source and appropriate powder particle distribution should be established to study the evolution mechanism of the melt pool and its affecting facts.

In this paper, FVM with VOF is applied to simulate the laser–powder interaction during the L-PBF process for Ti6Al4V based on a model with a combined heat source and Gaussian distribution of powder particle size. The model considers heat conduction, convection, evaporation and Marangoni force, and recoil pressure. Information on melt pool evolution, including temperature distribution and fluid flow, is obtained to study the formation mechanism of the geometry and surface morphology of the melt pool and their correlation. In particular, the melt pool evolution of the second laser scanning track is compared with that of the first track.

## 2. Simulation

### 2.1. Continuum Conservation Equations

The theoretical basis depends on the assumption of Newtonian, laminar, and incompressible flow, and their macroscopic continuum mass, momentum, and energy conservation equations can be described as follows:

(1) $$\frac{\partial \rho }{\partial t}+\nabla \cdot \left(\rho u\right)=0$$

(2) $$\frac{\partial \left(\rho u\right)}{\partial t}+\nabla \left(\rho uu\right)=-\nabla p+\nabla \cdot \left(\mu \nabla u\right)+f$$

(3) $$\frac{\partial \left(\rho C_{p}T\right)}{\partial t}+\nabla \cdot \left(\rho C_{p}Tu\right)=\nabla \cdot \left(\lambda \nabla T\right)+q$$

where ρ, u, p, μ, λ, $C_{p}$, and *T* are the density, velocity, pressure, dynamic viscosity, thermal conductivity, specific heat at constant pressure, and temperature, respectively. f is the body force per unit mass, and q is the energy from the heat source. It is worth noting that f includes the Boussinesq approximation:

(4) $$f_{b}=-\rho g\beta \left(T-T_{ref}\right)$$

which describes the buoyancy force caused by density differences that the Marangoni force, $\tau =-\frac{d\sigma }{\mathrm{dT}}\frac{dT}{dn}$, of which the surface tension changes with temperature gradient, applies only at the moving interface throughout the simulation process, and that recoil pressure, as a function of temperature *T*, is defined by

(5) $$P_{v}\left(T\right)=P_{0}\exp\;\left[\frac{\Delta H_{V}}{R_{g}}\left(\frac{1}{T_{V}}-\frac{1}{T}\right)\right]$$

In addition, deviatoric stress tensor [25]:

(6) $$\overset{=}{T}=2\mu \;\left[\left(\frac{1}{2}\nabla u+\frac{1}{2}{\left(\nabla u\right)}^{T}\right)-\frac{1}{3}\left(\nabla \cdot u\right)ΙΙ\right]$$

ΙΙ is the identified matrix.

### 2.2. Combined Rotating Volumetric Heat Source

**Figure 1:**
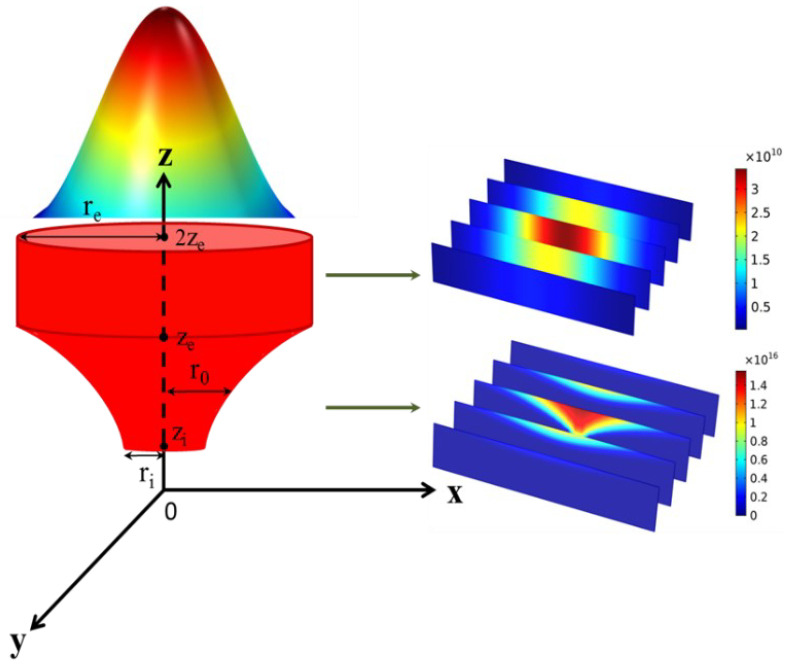
Schematic diagram of the heat source model and the interaction positions in the combined model. The upper part (parabolic rotating distribution volume heat source model) reveals the laser–powder interaction on the powder bed surface; the lower part (a cylindrical distribution Gaussian heat source model) reveals the laser–powder interaction inside the powder bed.

In the traditional simulation of laser–material interaction, a heat source is established to irradiate only on the material surface to be processed because the thickness (several millimeters) of the sample is significantly larger than the laser penetration depth. However, the thickness of the powder layer during the L-PBF process is normally less than 100 μm, but the laser penetration depth is normally larger than that. Due to the larger thickness of the laser penetration depth, an appropriate heat source should be established for the simulation of the L-PBF process. Furthermore, laser scattering occurs while irradiating on the surface of the powder bed, which causes the difference in laser energy distribution between the surface of and inside the powder bed. Therefore, an innovatively combined volumetric heat source rotating model to distinguish the difference in laser–powder interaction between the surface of and inside the powder bed is proposed. The heat source model and the interaction positions of the two heat sources in the combined model are schematically shown in Figure 1, consisting of a parabolic rotating distribution volume and a cylindrical distribution Gaussian heat source model. Its upper part reveals the laser–powder interaction on the surface of the powder bed, which can be described as follows [26]:

(7) $$Q{\left(x,y,z\right)}_{1}=\frac{3\eta P_{w}}{\pi r_{e}{}^{2}}\exp\left(-\frac{3\left({\left(x-vt-x_{0}\right)}^{2}+{\left(y-y_{0}\right)}^{2}\right)}{r_{e}{}^{2}}\right)$$

Its lower part reveals the laser–powder interaction inside the powder bed, which can be described as follows:

(8) $$Q{\left(x,y,z\right)}_{2}=\frac{3\eta P_{w}}{\pi \left(1-\exp\left(-3\right)\right)\left(A+B\right)}\left(\frac{1-\chi }{z_{e}-z_{i}}z+\frac{\chi z_{e}-z_{i}}{z_{e}-z_{i}}\right)\exp\left(-\frac{3\left({\left(x-vt-x_{0}\right)}^{2}+{\left(y-y_{0}\right)}^{2}\right)}{r_{0}^{2}\left(z\right)}\right)$$

$r_{0}$ is equal to:

(9) $$r_{0}\left(z\right)=\frac{z^{2}}{p}+s$$

*A* and *B* are described as:

(10) $$A=\frac{1-\chi }{z_{e}-z_{i}}\left[\left(\frac{1}{p^{2}}\frac{z_{e}^{6}}{6}+\frac{s}{p}\frac{z_{e}^{4}}{2}+\frac{s^{2}}{2}z_{e}^{2}\right)-\left(\frac{1}{p^{2}}\frac{z_{i}^{6}}{6}+\frac{s}{p}\frac{z_{i}^{4}}{2}+\frac{s^{2}}{2}z_{i}^{2}\right)\right]$$

(11) $$B=\frac{\chi z_{e}-z_{i}}{z_{e}-z_{i}}\left[\left(\frac{1}{p^{2}}\frac{z_{e}^{5}}{5}+2\frac{s}{p}\frac{z_{e}^{3}}{3}+s^{2}z_{e}\right)-\left(\frac{1}{p^{2}}\frac{z_{i}^{5}}{5}+2\frac{s}{p}\frac{z_{i}^{3}}{3}+s^{2}z_{i}\right)\right]$$

where χ is equal to 2, and $r_{i}=\frac{z_{i}{}^{2}}{p}+s$ and $r_{e}=\frac{z_{e}{}^{2}}{p}+s$ are brought into (10) and (11):

(12) $$p=\frac{z_{e}{}^{2}-z_{i}{}^{2}}{r_{e}-r_{i}}$$

(13) $$s=\frac{r_{i}z_{e}{}^{2}-r_{e}z_{i}{}^{2}}{z_{e}{}^{2}-z_{i}{}^{2}}$$

The depth of the melt pool during the L-PBF process is not as deep as that in the keyhole formed by deep laser penetration welding; thus, it is appropriate that $r_{i}=\frac{r_{e}}{2}$ and $z_{i}=z_{e}-r_{e}\ln2$.

### 2.3. Volume of Fluid (VOF)

In this paper, the VOF is used to trace the melt metal flow in the melt pool and the interface behavior between gas and liquid during the L-PBF process. When the evaporation of the metal is considered, the parameter for metal phase α_1_ and the gas phase α_2_ is introduced to describe the unit fluid, wherein α_1_ + α_2_ = 1. When α_1_ = 1, it means that all the volumes are metal phase. When α_1_ = 0, it means that all the volumes are gas phase. When 0 <α_1_ < 1, it means that there is a mixture of gas phase and melt metal phase inside the volumes. The evolution of the liquid–gas interface can be defined as:

(14) $$\frac{\partial \alpha_{1}}{\partial t}+\nabla \cdot \left(\alpha_{1}u\right)=-\frac{\dot{m_{v}}}{\rho_{2}}$$

In order to make the combined heat source model more compatible with the laser–powder interaction during the L-PBF process, this paper proposes the coefficient α_1_, which can be described as follows:

When 0 < α_1_ < 1:

$$Q_{\mathrm{source}}=Q\left(x,y,z\right)\times \alpha_{1}$$

when α_1_ = 0 or α_1_ = 1:

$$Q_{\mathrm{source}}=0$$

The “source”, namely the final heat source hitting the powder bed, ensures that laser energy has only an effect on the elements of the interface between the melt pool and gas in the surroundings.

### 2.4. Initial Condition and VOF for N-S and Energy Equation

The initial condition includes the initial temperature of the powder, substrate, and shielding gas of argon. Their initial temperature is equal to 400 K due to a preheating temperature of 100 ℃. Convection and radiation are considered on the top surface, while the other five surfaces are constant at a temperature of 400 K because their temperature change is negligible. The Navier–Stokes equation, which combines body forces and the VOF coefficient, is defined as [27]

(15) $$\frac{\partial \left(\rho u\right)}{\partial t}+\nabla \left(\rho uu\right)=-\nabla p+\nabla \cdot \overset{\cdot }{T}-\mathrm{Kc}\left(\frac{{\left(1-f_{l}\right)}^{2}}{f_{l}^{3}+C_{k}}\right)u+f_{b}+\left(\sigma \kappa n+\tau +n(p_{v}ΙΙ\cdot n\right)\left|\nabla \alpha_{1}\right|\frac{2\rho C_{p}}{\left(\rho_{1}C_{p1}+\rho_{2}C_{p2}\right)}$$

The total energy equation is given as

(16) $$\frac{\partial \left(\rho C_{p}T\right)}{\partial t}+\nabla \cdot \left(\rho C_{p}Tu\right)=-\frac{\partial \rho \Delta H_{f}}{\partial t}-\nabla \cdot \left(\rho u\Delta H_{f}\right)+\nabla \cdot \left(\lambda \nabla T\right)+\left\{Q_{\mathrm{source}}-\left[\sigma_{s}\epsilon \left(T^{4}-T_{\mathrm{ref}}^{4}\right)-h_{c}\left(T-T_{\mathrm{ref}}\right)\dot{+m_{V}}\Delta H_{V}\right]\left|\nabla \alpha_{1}\right|\right\}\frac{2\rho C_{p}}{\left(\rho_{1}C_{p1}+\rho_{2}C_{p2}\right)}$$

where $\sigma_{s}\epsilon \left(T^{4}-T_{\mathrm{ref}}^{4}\right)$ and $h_{c}\left(T-T_{\mathrm{ref}}\right)$ denote radiative and convective heat transfer between the powder layer and argon, respectively.$\;\dot{m_{V}}$ is the mass evaporation rate, defined as [28]:

(17) $$\dot{m_{V}}=P_{0}\exp\left\{\frac{\Delta H_{V}}{R_{g}}\left(\frac{1}{T_{V}}-\frac{1}{T}\right)\right\}\sqrt{\frac{M}{2\pi \mathrm{RT}}}$$

### 2.5. Model Establishment in Simulation

**Figure 2:**
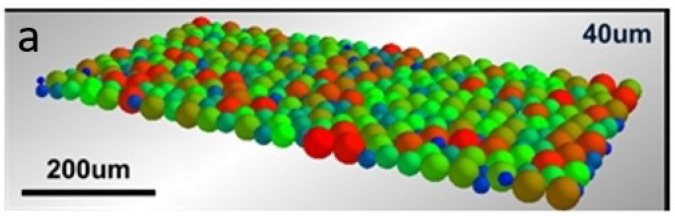

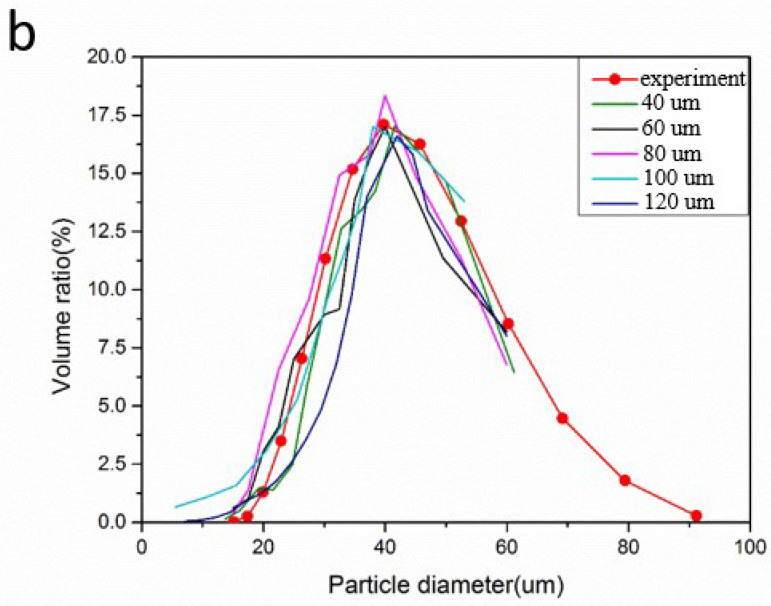
Geometric model of powder layer with a thickness of 40 μm (**a**); particle size distribution in simulation and that of actual powder particle size distribution in experiment (**b**).

The simulation of the laser–powder interaction with the powder bed model reflecting the complexity of the actual powder layer with particle packing can reproduce the surface morphology of the melt pool during the melt pool evolution, as well as the unsteady state due to the fluid flow in the melt pool during the L-PBF process. In this respect, this paper employed EDEM software for powder model establishment based on the true particle size distribution of Ti6Al4V, and then the information on the powder position was input into the model. The difference in densities of powder and gas was utilized to distinguish them. Figure 2 shows the geometric model of the powder layer with a thickness of 40 μm (Figure 2a) whose geometric size is 800 μm ×250 μm ×40 μm and the particle size distribution with layer thicknesses of 40, 60, 80, 100, and 120 μm in simulation and that of the actual powder particle size distribution in the experiment (Figure 2b). From Figure 2b, it can be seen that the experimental powder particle size distribution is similar to that in the simulation. Therefore, a layer thickness of 40 μm is reliable for simulation of the real power distribution.

**Figure 3:**
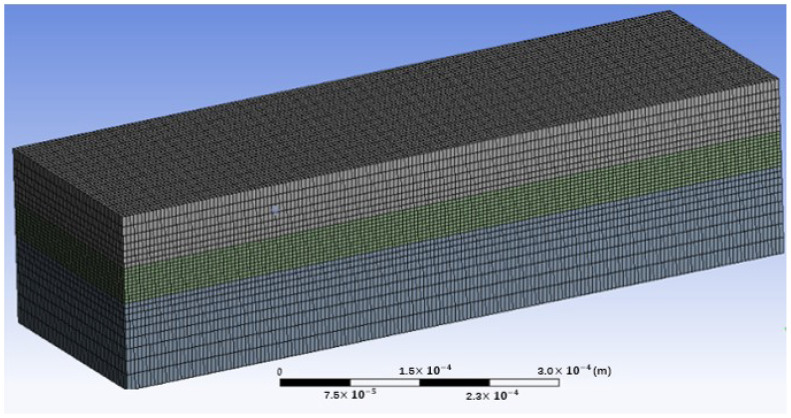
Meshing generation for simulation in laser–powder interaction.

**Figure 4:**
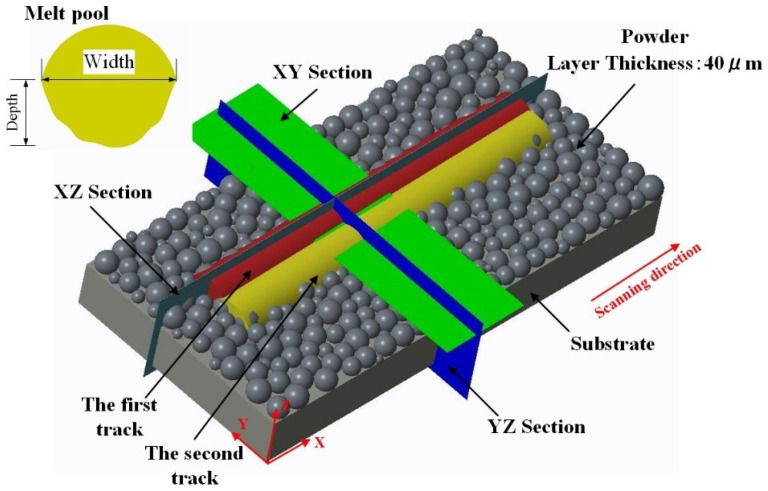
Simulation analysis model including powder layer, substrate, melt tracks and their laser scanning direction, different cross-sections, and melt pool geometries (depth and width).

Accuracy in numerical simulation is very important and primarily depends on the element and meshing with the appropriate models of the powder bed and the heat source. Figure 3 exhibits the meshing results, where hexahedral meshes are utilized for fine powder and coarse meshes for substrate due to the importance of the powder melt and resulting melt pool. The number of meshes and notes is 232,544 and 247,563, respectively, which is a balance between time, cost, and computing accuracy. To better understand the simulation model and the analysis of the melt pool characteristics, Figure 4 shows some important information, including the coordinate system of the model, the relative position of the powder layer with a thickness of 40 μm to the substrate, the laser scanning direction, the first and second melt tracks and their different sections, and the melt pool geometry (depth and width). The simulations in this paper were run on a desktop computer (2.5 GHz CPU and 16 GB RAM).

### 2.6. Thermophysical Properties of Material of Ti6Al4V

**Figure 5:**
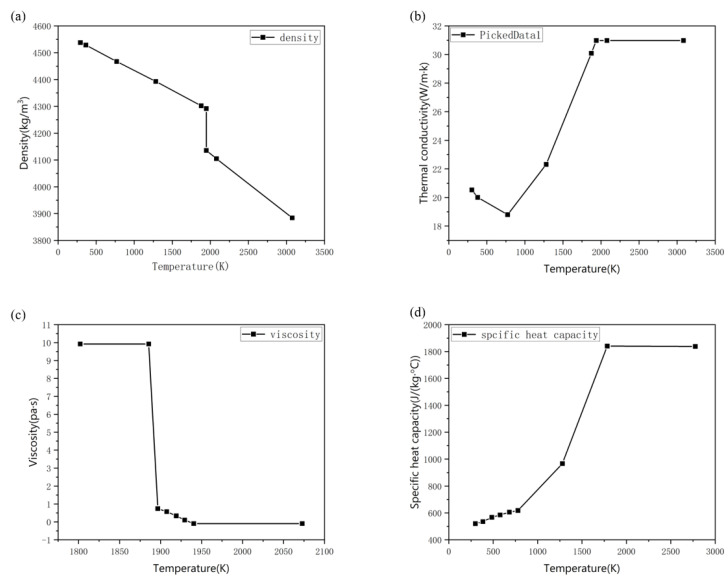
Thermophysical properties of Ti6Al4V and their temperature dependence (**a**). density; (**b**). thermal conductivity; (**c**). viscosity; (**d**). specific heat capacity.

In this paper, the multiphase flow model is used for numerical simulation. The model includes the gas phase of argon and the metal phase of Ti6Al4V. The simulation of the L-PBF process is a transient thermal analysis, in which the related thermophysical properties of the material such as specific heat capacity, density, viscosity, and thermal conductivity are temperature-dependent. The thermophysical properties of argon were selected from the parameter database provided in the software. In order to ensure computing accuracy in simulation, the thermophysical properties of Ti6Al4V change with the temperature, which is shown in Figure 5. Table 1 shows the data used for fluid flow and heat transfer in the simulation.

## 3. Experiments

**Figure 6:**
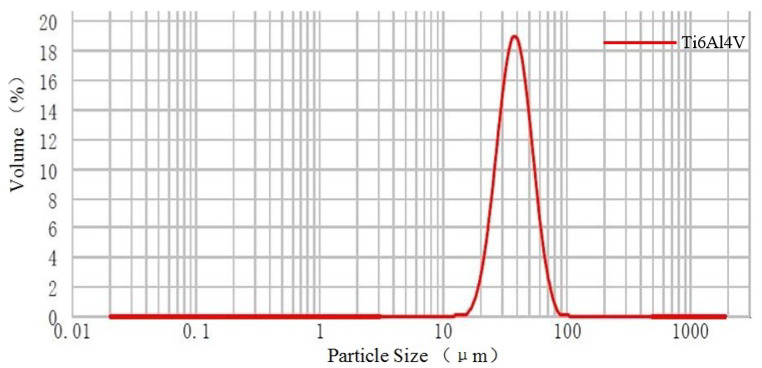
Particle size distribution of Ti6Al4V powder material.

**Figure 7:**
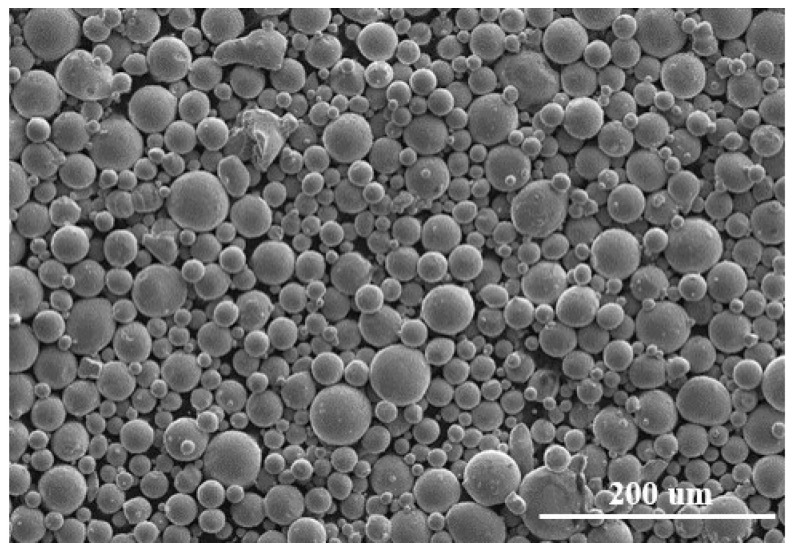
Morphology of Ti6Al4V powder material.

Gas-atomized Ti6Al4V powder material, of which the average particle size is 38 μm, was provided by EOS (EOS GmbH, Electro Optical Systems). Table 2 lists its nominal chemical composition. In addition, Figure 6 and Figure 7 reveal the size distribution of powder particles from 15 to 83 μm and their spherical morphologies in SEM, respectively. In order to avoid the possible formation of pores and the effect on the fluidity of the powder due to wet powder during the L-PBF process, the powder material should be dried under vacuum prior to the experiment.

The EOSINT M280 L-PBF machine was used for the experiment, of which the heat source was a YLR-400-SM continuous fiber laser provided by IPG with a wavelength of 1067 nm. Its peak value of output is 400 W with a beam diameter of about 100 μm. During the L-PBF process for Ti6Al4V alloy, the substrate should be preheated to 80 °C and flow with argon protection from possible oxidation and nitriding. In order to verify the results, the L-PBF process for single-track and double-track scanning was carried out using different process parameters in the experiment and simulation, respectively, which are shown in Table 3 in detail.

To observe the shape of the melt pool, the specimens were ground, polished, and then etched by Kroll etchant (25 mL HNO_3_, 15 mL HCl, 10 mL HF, 95 mL H_2_O) for about 30 s. However, the depth and width of the melt pool were not constant values during the single-track scanning. To truly reveal the morphology of the melt pool, one single track was measured by separation into several segments, and their mean value for the depth and width of the melt pool was obtained. Macrostructures were also observed via the OLYMPUS-DP72 optical microscope (OM) and measured by ImageJ.

**Figure 8:**
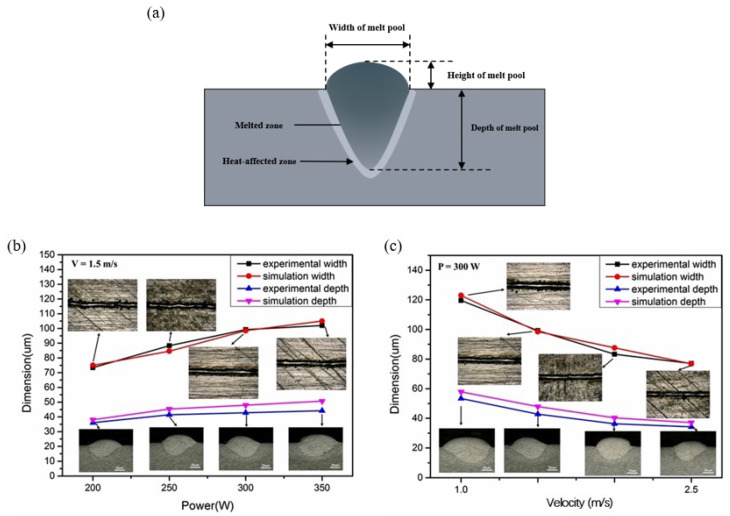
Schematic diagram of melt pool and its dimension (**a**); morphology and shape of the melt pool in experiment and simulation with different laser power (**b**); scanning velocity (**c**).

The consistency between the simulated and experimental results is very important, which means the simulation results are reliable, and the detailed information on the L-PBF process, including the temperature distribution and fluid flow in the melt pool, can be used to analyze the formation mechanism of the surface morphology and melt pool geometry. Figure 8 shows the change in the shape of the melt pool in the experiment and simulation with different laser power but constant scanning velocity (V = 1.5 m/s) (Figure 8b) and different scanning velocity (Figure 8c) but constant power (P = 300 W). The shapes of the melt pool are characterized by their depth and width in the simulations, as shown in Figure 8b,c, which is in good agreement with the experiments. The surface morphology of the scanning tracks with different process parameters is also shown in Figure 8b,c.

## 4. Results and Discussions

One of the important characteristics is the achievement of an L-PBF-processed component with almost 100% density. This means the current melt pool will metallurgically bond with adjacent scanning tracks and underlying solidified layers after its rapid solidification without any defects. In fact, Gunenthiram et al. [21] reported that when the laser irradiates on the powder bed during the L-PBF process, its process parameters such as laser power and scanning velocity collectively determine the heat input, which causes the formation of the melt pool with specific geometry and surface morphology and further the specific temperature distribution and fluid flow in the melt pool. With the movement of the laser beam, the melt pool cools down, the specific geometry and surface morphology of the melt pool change with the cooling process, and a solidified track forms continuously. According to Kusuma et al. [26], the specific geometry and surface morphology of the melt pool are important indicators of the interaction between the temperature distribution and fluid flow. Their interaction mechanism and the effect on the melt pool evolution should be investigated in detail.

### 4.1. Evolution of the Melt Pool and Its Affecting Factors

#### 4.1.1. Geometry and Surface Morphology of the Melt Pool

**Figure 9:**
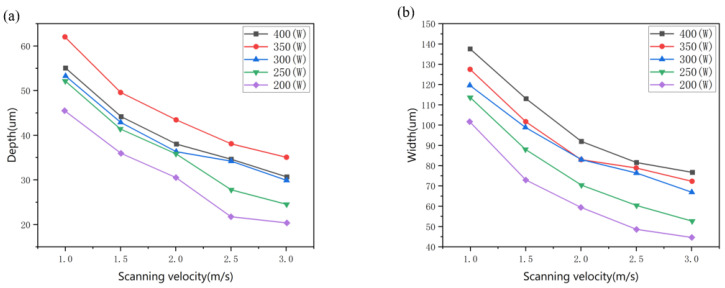
Geometry change of the melt pool characterized by the depth (**a**) and width (**b**) with different laser power and scanning velocity.

The curves in Figure 9a,b reveal the geometry (characterized by the depth and width of the melt pool) changing with laser power and scanning velocity. It is observed the depth and width of the melt pool decrease with the increase in scanning velocity when the laser power remains constant. When the scanning velocity is kept constant, they become deeper and wider with the increase in laser power. When the scanning velocity is 1000 mm/s, the depth increases from 46 μm to 61 μm, while the laser power increases from 200 to 400 W, as shown in Figure 9a, which is more than 1.5 times the layer thickness. A deeper remelting zone more than the layer thickness provides the achievement of a possible denser metallurgical bonding. When the laser scans at a higher velocity of 2000 mm/s, shallower depths about 30 μm to 38 μm are achieved, while when the laser power increases from 200 to 400 W, the heat source is not sufficient for the formation of a denser bonding with the underlying layer. Figure 9b shows the width of the melt pool changing with laser power and scanning velocity, which tends to be similar to the depth.

**Figure 10:**
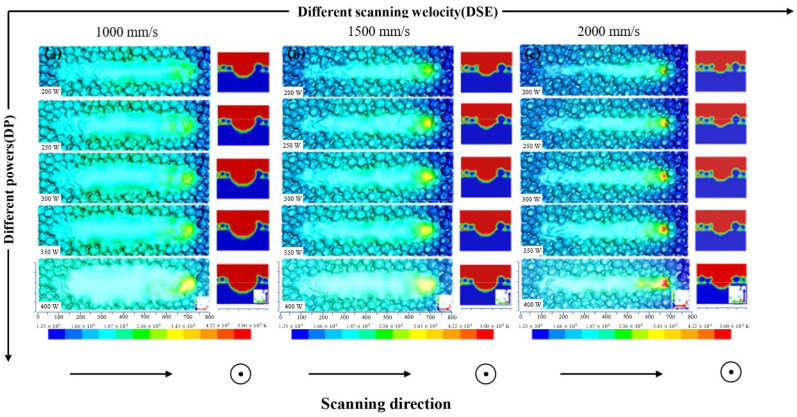
Influence of process parameters with laser power from 200 W to 400 W and scanning velocity from 1000 mm/s to 2000 mm/s on the melt pool geometry (in top view) and surface morphology (in section view).

Figure 10 exhibits the change in the melt pool geometry and surface morphology with laser power and scanning velocity (only part of the process parameters in Figure 9). It is obvious that the melt pool becomes wider and deeper with the increase in laser power, which is shown on the different power (DP) axis. However, the tendency is the opposite on the different scanning velocity (DSV) axis. The reason is that when the laser power is constant, the interaction time between the laser beam and the powder material becomes shorter as the scanning speed increases, resulting in a decrease in the heat input, which further reduces the width and depth of the melt pool. However, the increase in the heat input leads to an increase in the depth and width of the melt pool as the laser power increases when the scan speed is constant. The surface morphology shows the appearance of the melt track, the wetting condition between the melt track, and surrounding powder particles. From our experience with the L-PBF process of Ti6Al4V alloy, a laser power of 300 W and a scanning velocity of 1500 mm/s are appropriate process parameters for component fabrication using the L-PBF process. Compared with the surface morphology fabricated using the L-PBF parameters, it appears that with the appropriate melt metal on the powder bed, the width of the melt track is 2 or 3 times the average powder particle size, and there are several fish-scale patterns at the beginning of the melt track. When the laser power is 400 W and the scanning velocity is 1000 mm/s, the morphology of the melt pool appears much smoother, more powder particles are drawn into the melt track, and further, there is much more melt metal on the powder bed, which means excessive heat input. When the laser power is 200 W and the scanning velocity is 2000 mm/s, the surface of the melt is rugged, there is less melt metal, and the largest powder particle diameter is even larger than the width of the melt track, which means too little heat input.

#### 4.1.2. Fluid Flow of the Melt Pool

**Figure 11:**
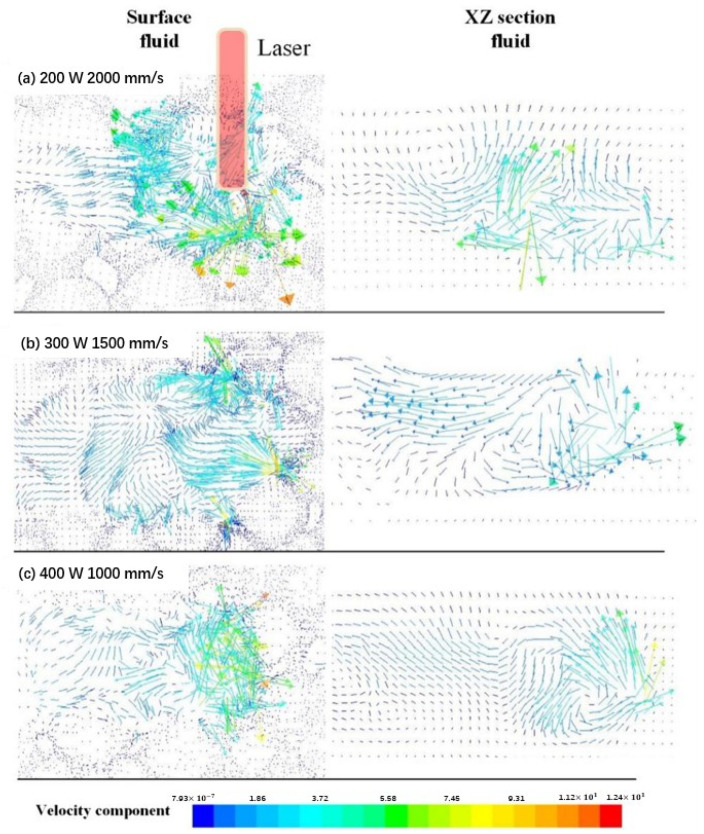
Thermofluid of the melt pool at the time of 200 μs in top view and XZ section view with process parameters of 200 W-2000 mm/s (**a**), 300 W-1500 mm/s (**b**), and 400 W-1000 mm/s (**c**).

Based on the fluid-dynamic mechanism in mesoscale, the depth and width dependent on its temperature distribution are the results of the fluid flow in the melt pool, which is collectively determined by process parameters such as laser power, scanning velocity, and so on. Figure 11 shows the fluid flow in the melt pool at the laser scanning time of 200 μs with three sets of process parameters.

When the laser power is 200 W and the scanning velocity is 2000 mm/s, the surface fluid flows only in a narrow space with a higher velocity. Its flow direction is chaotic due to the obstruction of the surrounding unmelted powder particles in Figure 11a and the shallower melt pool shown. What causes the rugged surface morphology in front of the melt track and possibly worse bonding with the substrate is the not completely melted powder particles caused by the lower heat input. This is similar to the formation of rough surface morphology and discontinuous melt pool geometry shown in Figure 10 by p = 200 W and v = 2000 mm/s, according to Panwisawa et al. [27,28], which is the possible source of defects such as holes and balling.

When the laser power is 300 W and the scanning velocity is 1500 mm/s, as shown in Figure 11b, the keyhole forms at the center of the melt pool, while the fluid flows in the bottom-up direction and then from center to back to front of the keyhole formation. Affected by the Marangoni force, the fluid appears continuous and steady, which means better wetting between the melt metal and surrounding powder particles.

When the laser irradiates on the powder bed with a higher laser power of 400 W and a relatively slower velocity of 1000 mm/s, the temperature of the fluid in the melt pool is at a relatively higher level. Its flow direction is not obvious, as can be seen in Figure 11c. However, according to Gunenthiram et al. [21], higher fluid velocity and the more violent evaporation associated with a higher heat input will lead to another possible problem such as spatter.

### 4.2. Melt Pool Evolution and Its Temperature Fluctuation Mechanism of the First Scanning Track

#### 4.2.1. Melt Pool Characteristic at the Initial, Intermediate, and End Positions of Laser Scanning Process

As mentioned above, when the laser irradiates on the powder bed with a laser power of 300 W and a scanning velocity of 1500 mm/s, there are better wetting conditions between the melt metal and powder particles, and a continuous and steady fluid flow in the melt pool during the L-PBF process forms. Therefore, this parameter is chosen as an example to understand the change in melt pool characteristics with time, i.e., the evolution of the melt pool. It is well known that melt pool geometry depends on the melt fluid determined by the temperature gradient, which is determined by the process parameters. The temperature gradient of the melt pool is always changing with time due to the heat accumulation during the L-PBF process; thus, the corresponding fluid flow and melt pool geometry are also changing.

**Figure 12:**
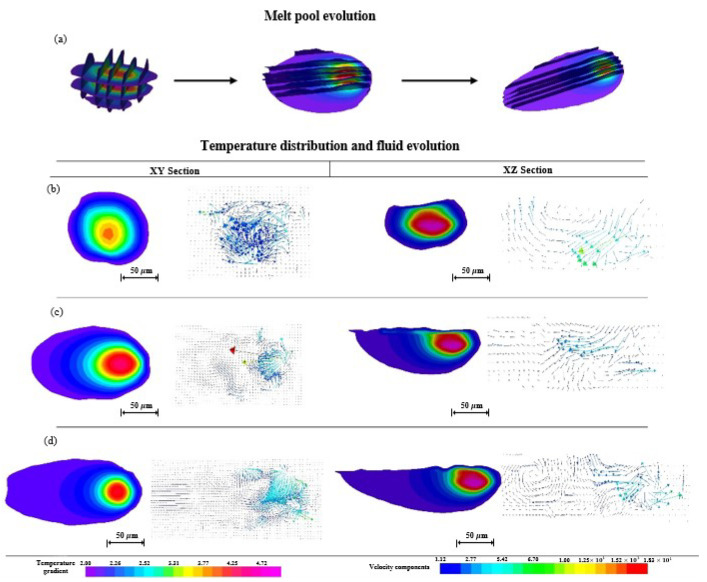
Evolution of melt pool geometry (**a**); evolution of temperature gradient and fluid flow at initial (**b**), intermediate (**c**), and end (**d**) positions of single scanning track in XY and XZ sections, respectively.

Figure 12a shows the evolution of the melt pool during the single-track scanning process at the initial, intermediate, and end positions using the melt pool geometry in quasi-3D. The melt pool geometry with a boundary temperature of 1928 K (the melting point of Ti6Al4V) is cut with three XY sections and five XZ sections to show in detail the melt pool geometry, surface morphology, temperature gradient, and fluid flow in different positions of the melt pool. The melt pool geometry changes from approximately circular, elliptical, to a “comet” shape with time, where the width and depth at the intermediate and end positions are larger than that at the initial position.

Figure 12b–d shows the evolution of the temperature gradient and fluid flow at the initial, intermediate, and end positions of the single-track scanning in their XY and XZ sections, respectively, which are obtained by cutting with the intermediate planes of the melt pool. The temperature gradient and fluid flow in the XY section are the top views, while those in the XZ section are side views. The temperature in the melt metal at the initial position (Figure 12b) is relatively lower. Its melt metal flows in all directions in a narrow space because it is surrounded by non-melt powder particles, and it is difficult to wet them. The fluid flow in the XZ section flows with a “U”-shape route, which is affected by the Marangoni force and the limited melt space mentioned above. The higher temperature is located in the lower right corner. The powder particles near the high-temperature zone are completely molten and flow in the bottom-up direction. However, the heat in the melt metal dissipates through thermal conduction to the powder bed, and the fluid is not able to flow to the deeper zone. The flow direction changes upward and solidifies gradually. It should be noted that the flow velocity of the fluid is several meters per second.

The melt pool becomes deeper and longer with the continuous laser scanning process. There is more space for the melt metal to flow and make it possible to form a continuous and steady flow in the opposite direction to the laser scanning (Figure 12c,d). Furthermore, the temperature gradient and fluid flow in the XZ sections in Figure 12 show the melt metal flows steadily only for a short period of time and then moves like a vortex, which causes the surface of the melt pool to not be smooth.

#### 4.2.2. Temperature Fluctuation and Its Mechanism

**Figure 13:**
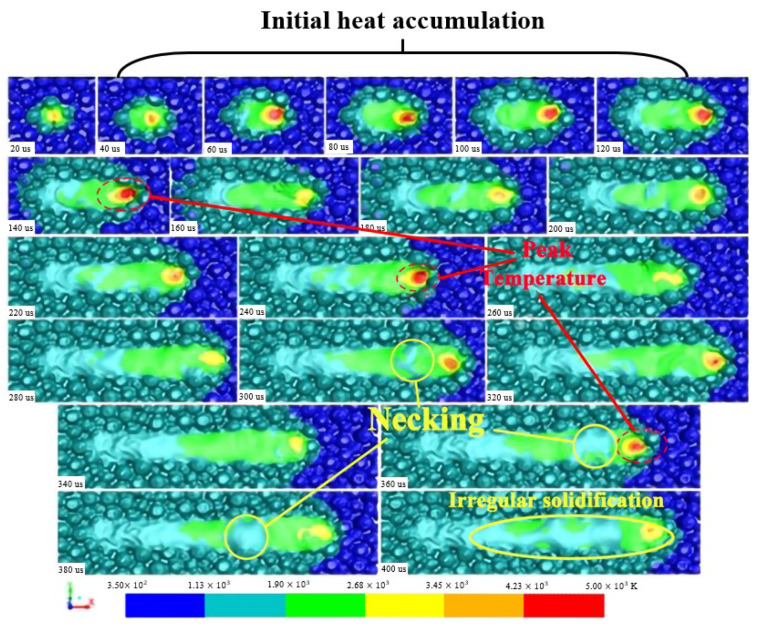
Change in temperature distribution and surface morphology of the melt pool of one single-track scanning with time.

Figure 13 shows the change in the temperature distribution and surface morphology of the melt pool during one single-track scanning within 400 μs. The temperature in the melt pool in the initial position is relatively lower, increasing to a peak temperature of about 5000 K and in only about 80 μs (from 60 μs to 140 μs). It then decreases again to about 3400 K, which is about the evaporation temperature. However, as the laser scanning process progresses, the temperature in the melt pool reaches about 5000 K again, but it cannot remain longer than 100 μs. Because the temperature in the melt pool fluctuates with the laser scanning process, its period is about 100 μs. Its fluctuation mechanism is revealed by the fluid flow in the melt pool, causing fast heat diffusion.

As reported by Khairallah et al. [16], there is often a small area in the simulated melt track whose temperature is slightly lower than the melting point, and its width is narrower than that of the melt pool during the melt pool evolution called necking. If the necking cannot be bridged by the tail of the solidified area, which separates the melt pool into several parts and thus forms irregular solidification, it results in a not solidified melt track from the tail to the front of the scanning track providing more solidification direction. Fortunately, the necking in the melt track processed by the process parameters of P = 300 W and v = 1500 mm/s is bridged by solidified melt because of its continuous and steady fluid flow.

**Figure 14:**
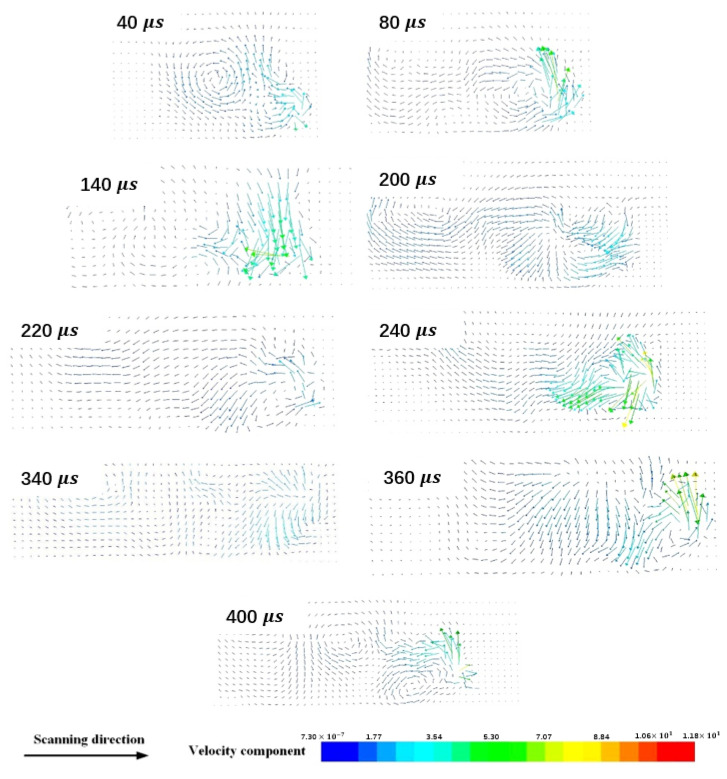
Change in fluid flow in the melt pool of one single-track scanning in XZ section with time.

What causes the temperature fluctuation during the melt pool evolution of one single-track scanning? The fluid flow in the melt pool shown in Figure 14 is used to reveal the mechanism of the temperature fluctuation of the melt metal. It is well known that the initial temperature in the melt pool surrounded by powder particles is relatively lower, and its thermal conductivity is also lower than that of the solidified metal having the same dimension. It is difficult to establish efficient heat dissipation between the melt metal and the surroundings, although the transient fluids at the times of 40 μs and 80 μs shown in Figure 14 flow violently in the melt pool due to the possible shorter time duration. The temperature in the initial heat accumulation area (IHAA) reaches a peak value and lasts for the longest time during the single-track scanning process. The peak temperature in the IHAA is the result of the interaction between the thermal conduction and convection in the melt pool under the condition of the initial phase of the laser irradiating on the powder materials.

The formation mechanism of the peak temperature in the IHAA is the same as that at other positions. The peak temperature occurs at the moments of 140 μs, 240 μs, and 360 μs during the single-track scanning, respectively, and their corresponding flow velocity reaches about 8000 mm/s, i.e., the fluid flow with the highest flow velocity. At that time, the melt metal (fluid) no longer flows backwards, the vortex forms in front of the melt pool, and the direction of fluid flow behind the melt pool is not obvious. The fluid with a higher flow velocity in the front end of the melt pool brings the melt metal with a high temperature to the opposite direction of the laser beam scanning, and the temperature at the beam center will decrease. However, the fluids at moments of 120 μs, 220 μs, and 340 μs do not flow violently as those at moments of 140 μs, 240 μs, and 360 μs, respectively. They flow backward with a continuous and steady flow with a flow velocity of about 3500 mm/s. A slower flow velocity causes a gradual increase in the temperature of the melt metal at the beam center. The formation mechanism of the peak temperature is attributed to the heat accumulation with time, and its interval is about 100 μs.

The formation mechanism of the necking is related to the fluid flow, too. The peak temperature at the beam center causes an upward fluid flow, resulting in an unsmooth surface of the melt pool due to much more efficient heat dissipation between the top surface of the melt pool and the surroundings, which further leads to necking. Therefore, melt solidification begins both on the top and bottom of the melt pool, as mentioned by Basak et al. [29].

### 4.3. Melt Pool Evolution of the Second Scanning Track

#### 4.3.1. Melt Pool Characteristic of the Second Scanning Track

The second scanning track is different from the first in terms of its temperature gradient, fluid flow in the melt pool, and melt pool geometry. The melt pool of the second scanning track is a powder bed on one side and solidified metal on the other side. The temperature in the melt pool is relatively higher due to heat accumulation in the first track, and there is an overlapping zone between the two tracks. The comparison of the melt pool evolution between the first and second tracks using the L-PBF process by the parameters P = 300 W and V = 1500 mm/s benefits the understanding of the influence of the temperature distribution and fluid flow on the surface morphology.

**Figure 15:**
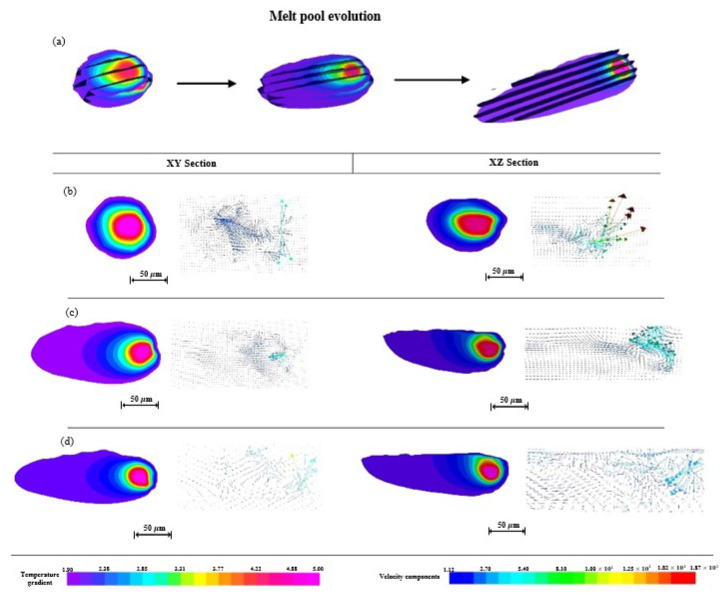
Evolution of the melt pool geometry (**a**); evolution of temperature gradient and fluid flow at initial (**b**), intermediate, (**c**) and end (**d**) position of the second track scanning in XY and XZ sections, respectively.

The melt pool of the second track becomes deeper and wider with the continuation of laser scanning, whose evolution trend is similar to that of the first scanning. Figure 15 exhibits the evolution of the melt pool geometry (Figure 15a), the evolution of the temperature gradient, and the fluid flow at the initial (Figure 15b), intermediate (Figure 15c), and end (Figure 15d) positions of the second-track scanning in the XY and XZ sections, respectively. However, the geometry of the melt pool changes from approximately circular, elliptical, to a “comet” shape of the second track shown in Figure 15, whose three-dimensional size is larger than that of the first track in Figure 12. This is attributed to the higher temperature level in the melt pool of the second track due to the heat accumulation during the first track scanning. The area inside the isothermal line of the liquid–solid boundary of the second track is larger than that of the first track, and not only is the temperature far higher than 1928 K, but the temperature of the powder bed is also increased due to the heat accumulation during the first track scanning.

In terms of the characteristics of fluid flow in the melt pool of the second track, it flows more violently at the initial position, its velocity becomes faster, and the direction becomes more disordered. The flow velocity arrows in the XY section show that the fluid on the top surface is steadier than that of the first track, and it has no tendency to form vortex flow. The outline of the melt pool in the XZ section is smoother than that shown in Figure 12, which suggests the steady fluid flow in the melt pool of the second track.

#### 4.3.2. Evolution Mechanism of the Second Track

During the L-PBF processing of the second scanning track using the same process parameters, it has a different thermal conduction environment than that of the first track. Its fluid flow in the melt pool in the initial position is violent and temperature-dependent, and the temperature of the second track on the powder bed is about 500 K higher than that of the first. Higher temperature leads to higher surface tension and a more violent Marangoni effect because both of them have a positive correlation with temperature. The temperature in the melt pool is higher than that on the surface of the melt pool due to the efficient thermal conduction of the surface, and the fluid with higher energy is able to produce a bigger melt pool due to the higher energy input. Moreover, the fluid flow in a “U” shape is similar to the initial position of the first track. At the intermediate and end positions of the second track, the melt pool size becomes larger due to the heat accumulation, and the temperature is at a higher level, creating a bigger space for the formation of a steady fluid flow.

The edge of the melt pool of the second track near the powder side is not as smooth as the other side, suggesting that the powder particles cause the roughness of the side surface of the melt pool. The powder particles are sintered due to the lower temperature than the melting point, but it is enough for bonding with the melt pool in a not fully melted condition. However, one side near the first track is the edge of the remelting metal, and its surface quality is better.

In summary, the temperature distribution and the thermal dynamics in the melt pool of the first track are different from those of the second. The main reason for their differences is that the heat accumulation generated during the fabrication process of the first melt pool will affect the formation of the second, and the temperature increase caused by the heat accumulation of the first track will also affect the formation of the second melt pool. Increasing the laser absorption rate of the powder materials brought from the heat accumulation affects the temperature gradient and melt flow in the melt pool, which further affects the size of the melt pool and the process of liquid metal crystallization. On the other hand, the thermal conductivity of the TC4 material is low, and the heat accumulation is more serious than that of the material with good thermal conductivity. From the macroscopic view of the material and temperature accumulation in the additive manufacturing process, the above rules can represent the entire L-PBF-fabrication process. Microscopically, the temperature gradient and heat flow in each melt pool are different, and the crystallization of the liquid metal is different, resulting in different microstructural properties at different positions inside the L-PBF-fabricated part.

## 5. Conclusions

In this paper, a physical model with VOF in FVM is established to trace the free interface of the melt pool during the L-PBF-fabricated Ti6Al4V process. Only the loading in the unit interface between the metallic and gas phase in the laser–powder interaction zone is carried out by a combined heat source. The simulation results help to analyze the melt pool evolution mechanism by providing information on the fluid flow and temperature distribution in the melt pool, determined by different process parameters. The following conclusions can be drawn:

- The melt pool geometry, characterized by the width and depth of the melt pool, is determined significantly by laser power and scanning velocity. However, its width and depth change with the increase in scanning velocity from 1.5 m/s to 2.0 m/s and laser power from 250 W to 350 W;
- The melt pool geometry of the first track changes from approximately circular, elliptical, to a “comet” shape because of the thermal accumulation during the continuous laser scanning process with a laser power of 300 W and a laser scan velocity of 1.5 m/s. Due to the keyhole formation at the center of the melt pool, the fluid flows in a bottom-up direction and then from center to back. Affected by the Marangoni force, the fluid appears continuous and steady.
- During the L-PBF process, the geometry of the melt pool of the second track becomes larger due to the heat accumulation during the continuous laser scanning process. The melt pool of the second track flows violently in the initial position due to the higher flow velocity in the limited melt space affected by the Marangoni effect and recoil pressure. A sufficiently long melt pool helps to obtain a steady fluid and smooth surface morphology. Thus, the second track has enough space for metal flow, and it is more likely to obtain smooth surface morphology than the first track.
- The evolution trend in thermal dynamics of the second track in the melt pool is similar to that of the first track, but its geometry is larger, its temperature is higher, the fluid flow is faster, and the surface of the melt pool is much smoother than that of the first track.
- The temperature in the melt pool in the beam center fluctuates during the laser scanning process, which is possibly attributed to the interaction between thermal conduction and violent fluid flow or the interaction between heat accumulation and violent fluid flow.

## Figures and Tables

**Table 1:** Material parameters setting for simulation in fluid flow and heat transfer.

Physical Quantities	Ti6Al4V
Solidus temperature	1878 (K)
Liquidus temperature	1928 (K)
Evaporation temperature	3133 (K)
Molar mass	46.49 (g/mol)
Viscosity	0.005 (pa·s)
Surface tension	1.4 (N/m)
Temperature coefficient of surface tension	−0.26 × 10^−3^ (N/m·K)
Thermal expansion coefficient	8 × 10^−6^ (K^−1^)
Atmospheric intensity of pressure	101,300 (N/m^2^)
Ideal gas constant	8.314 (J/K·mol)
Boltzmann constant	1.38 × 10^−24^ (J/K)

**Table 2:** Nominal chemical compositions of gas-atomized powder material of Ti6Al4V alloy (wt%).

Element	Ti	Al	V	O	N	C	H	Fe
**powder**	Balance	5.5–6.75	3.5–4.5	0.2	0.05	0.08	0.015	0.3

**Table 3:** Process parameters of L-PBF process.

	Laser Power (W)	Scanning Velocity (m/s)	Thickness (μm)	Hatch Space (μm)
First scanning track	200, 250, 300, 350,400	1.5	40	100
	300	1.0, 1.5, 2.0, 2.5, 3.0	40	100
Second scanning track	300	1.5	40	100

## Data Availability

The data presented in this study are available from the corresponding authors upon reasonable request.

## Nomenclature

ρ	Material density, kg/m^3^
V	Velocity vector, m/s
P	Pressure, Pa
F	Volume force, N
μ	Viscosity, Pa·s
$C_{P}$	Specific heat capacity, J/(kg·K)
ΔH	Phase change of latent heat, J
Κ	Thermal conductivity, W/(m·K)
$\alpha_{1}$	Metal phase coefficient
$\alpha_{2}$	Gas phase coefficient
$\Delta H_{V}$	Evaporation enthalpy change, J
R	Ideal gas constant
$R_{g}$	General gas constant
*u, v, w*	Velocity component, m/s
g	Gravity acceleration, m/s^2^
$m_{V}$	Evaporation rate per volume
$P_{w}$	Laser power, W
θ	Porosity ratio
η	Laser absorption coefficient
P	Laser power, W
$z_{i}$	Coordinate of lower heat source
$r_{i}$	Radius of lower heat source, μm
χ	Heat flow peak proportional coefficient
v	Scanning velocity, mm/s
$z_{e}$	Powder thickness, μm
$r_{e}$	Beam spot radius, μm
$h_{c}$	Convective heat transfer coefficient
$\sigma_{s}$	Boltzmann constant
ϵ	Radiative coefficient
Q	Laser heat flux density, J/(m^2^·s)
$P_{0}$	Atmospheric pressure, Pa
